# Atrial Fibrillation, Oral Anticoagulants, and Concomitant Active Cancer: Benefits and Risks

**DOI:** 10.1055/s-0041-1728670

**Published:** 2021-06-01

**Authors:** Adriano Atterman, Leif Friberg, Kjell Asplund, Johan Engdahl

**Affiliations:** 1 Department of Clinical Sciences, Karolinska Institutet, Danderyd Hospital, Stockholm, Sweden; 2 Department of Public Health and Clinical Medicine, Umeå University, Umeå, Sweden

**Keywords:** atrial fibrillation, stroke prevention, cancer

## Abstract

**Aim** To determine to what extent active cancer influences the benefit–risk relationship among patients with atrial fibrillation receiving oral anticoagulants for stroke prevention.

**Methods** In this cohort study of all patients with atrial fibrillation in the Swedish Patient register during 2006 to 2017, 8,228 patients with active cancer and 323,394 without cancer were followed up to 1 year after initiation of oral anticoagulants. Cox regression models, adjusting for confounders and the competing risk of death, were used to assess risk of cerebrovascular and bleeding events.

**Results** Among patients treated with oral anticoagulants, the risk for cerebrovascular events did not differ between cancer patients and noncancer patients (subhazard ratio [sHR]: 1.12, 95% confidence interval [CI]: 0.98–1.29). Cancer patients had a higher risk for bleedings (sHR: 1.69, CI: 1.56–1.82), but not for fatal bleedings (sHR: 1.17, CI: 0.80–1.70). Use of nonvitamin K oral anticoagulants was associated with lower risk of both cerebrovascular events and bleedings compared with warfarin.

**Conclusion** Patients with atrial fibrillation and active cancer appear to have similar net cerebrovascular benefit of oral anticoagulant treatment to patients without cancer, despite an increased risk of nonfatal bleedings. Use of nonvitamin K oral anticoagulants was associated with lower risk of all studied outcomes.

## Introduction

Patients with cancer have increased risk of bleeding, which can be a problem when considering oral anticoagulants (OACs) for stroke prevention in patients with atrial fibrillation (AF). 1 Current AF guidelines do not specifically address OAC treatment among cancer patients 2 3 ; however, previous studies indicate that cancer patients with AF benefit from being treated with OACs. 4 5

The main goal with OAC treatment in patients with AF is to prevent ischemic stroke, balancing the increased risk of bleedings, especially intracranial, which has high mortality and the potential to cause impairment of function and life quality, as well as rising health and social care costs. 6 7

In a previous study of AF patients, we found net cerebrovascular benefit (defined as reduced risk of ischemic stroke as well as intracranial bleeding) with OAC treatment compared with no OAC treatment among both cancer and noncancer patients analyzed separately. In the present study, our aim was to study how active cancer influences the net cerebrovascular benefit and bleedings after initiation of OAC treatment in patients with AF.

## Methods

### Study Design and Data Source

In this retrospective cohort study, cross-linking Swedish health registers, all individuals with a diagnosis of AF between January 1, 2006 and December 31, 2017 were identified from the National Swedish Patient Register. Patients aged <18 or >100 years and patients with an absolute indication for OAC due to diagnosis of mitral stenosis or mechanical heart valve were excluded.

### Registers

The National Swedish Patient Register has shown positive predictive values for AF and stroke of 97 and 88%, respectively, and of 85 to 95% for other diagnoses including bleedings. 8 9 10 11 The Cancer Register is prospective, has a completeness of 96%, and holds information on, e.g., tumor site. 12 The Drug Register provides information on all dispensed prescription drugs in Sweden since 2005. 13 The Cause of Death Register contains details about all deaths which have occurred in the country, and its completeness is high. 14

### Definitions

AF-related OAC initiation was defined by the first dispensing of OAC adjacent to the first registered AF diagnosis during the study period: at the earliest 6 months before the AF diagnosis and at the latest December 30, 2017. OACs were subgrouped into warfarin (the only registered vitamin K antagonist in Sweden) and nonvitamin K OACs (NOACs). Information on drug dispensation was collected from the Drug Register. Treatment was defined as at least one dispensed OAC prescription.

Information on comorbidity at OAC initiation was collected from the Patient Register using information from 1997 onwards, when the International Classification of Diseases 10th Revision was implemented in Sweden ( Supplementary Table S1 , available online only).

Patients with cancer were restricted to those with active cancer defined as a new cancer diagnosis other than basalioma registered within 1 year prior to OAC initiation in either the Patient or the Cancer Register, not preceded by any cancer diagnoses during the 5 years before OAC initiation. Noncancer patients were defined as individuals without any cancer diagnosis in the previous 5 years. The presence of alcohol-related disease was assessed with a composite of codes used by the Swedish Board of Health and Welfare for estimating alcohol-related deaths. The stroke risk score CHA _2_ DS _2_ -VASc 15 was used without counting points for female sex, and the bleeding risk score HAS-BLED 16 without counting points for labile prothrombin time or international normalized ratio.

Time at risk was calculated as within 1 year from OAC initiation to first event of interest, emigration, death, or end of follow-up (December 31, 2017). The composite endpoint cerebrovascular events comprised ischemic stroke and intracranial bleedings. Bleeding was defined as an admission to a hospital with a major or nonmajor clinically relevant bleeding diagnosis as described in Supplementary Table S1 , available online only. For ischemic strokes, only the primary or secondary diagnosis code position was considered, whereas for bleedings, any position was considered. 11

### Statistical Methods

Descriptive data are presented as means or proportions. Differences between groups are described with standardized differences, and incidence rates as events per 100 patient-years.

In multivariable Cox regression analyses, we included heart failure, hypertension, age, diabetes, the composite prior ischemic stroke/transient ischemic attack/peripheral arterial emboli, vascular disease, sex, year of OAC initiation, NOAC (instead of warfarin) treatment, prior intracerebral bleeding, impaired kidney function, frequent falls, anemia, prior major bleedings, liver disease, and alcohol-related disease, depending on the outcome event of interest. All analyses were conducted taking the competing risk of death before an endpoint event into account: cumulative incidences were estimated with the Aalen–Johansen nonparametric method, 17 and multivariable subhazard ratios (sHRs) were computed according to the method of Fine and Gray. 18 Tests were two-sided and used 95% confidence intervals (CIs). *p* -Values <0.05 and standardized differences >10% were considered significant.

Analyses were performed using Stata version 15.1 (StataCorp, College Station, Texas, United States).

### Ethics

The study conforms to the Declaration of Helsinki and was approved by the regional ethics committee (EPN 2018/1252–31). Individual patient consent was not required or obtained.

## Results

### Patient Characteristics

As presented in Table 1 , the study population of AF patients consisted of 8,228 patients with active cancer and 323,394 patients without cancer, all of whom had been started on OAC treatment. The proportions of warfarin and NOAC users did not differ significantly between cancer and noncancer patients. Cancer patients were more often male, older, and had higher CHA _2_ DS _2_ -VASc and HAS-BLED scores. They also more often had a history of anemia, gastrointestinal bleedings, and venous thromboembolism. The most common cancer type was urological cancer, followed by gastrointestinal, hematological, breast, lung, gynecological, and intracranial cancers.

**Table 1:** Patients with atrial fibrillation, cancer versus noncancer: baseline data at OAC initiation

	At OAC initiation		
	Cancer	Noncancer	Standardized difference
*N* (%)	8,228 (2.5%)	323,394 (97.5%)	
Female	36.5%	43.3%	**0.139**
Age (mean)	75.1	73.1	**−0.211**
Age distribution			
< 65 y	10.3%	19.5%	**0.266**
65–74 y	35.3%	32.1%	
75–84 y	41.6%	35.5%	
> 84 years	12.7%	12.9%	
Year of OAC initiation			
2005–2011	46.6%	52.8%	**0.123**
2012–2017	53.4%	47.2%	
Risk scores at OAC initiation			
CHADS2-VASc (mean)	3.0	2.8	**−0.129**
Low (0 points)	3.9%	8.5%	**−0.211**
Intermediate (1 point)	14.3%	16.0%	
High (2–8 points)	81.9%	75.5%	
HAS-BLED (mean)	2.3	2.0	**0.188**
Low (0–1 points)	28.2%	36.4%	**0.189**
Intermediate (2 points)	30.7%	29.6%	
High (3–5 points)	40.7%	33.6%	
Very high (>5 points)	0.5%	0.3%	
Comorbidity at OAC initiation			
Heart failure	24.0%	22.7%	0.031
Hypertension	55.3%	49.7%	**0.113**
Ischemic heart disease	26.4%	26.5%	0.003
Prior PCI	7.8%	7.9%	0.005
Diabetes	17.2%	15.6%	0.042
Impaired kidney function	5.4%	3.6%	0.086
End renal stage/dialysis	0.4%	0.3%	0.018
Prior ischemic stroke	11.9%	13.0%	0.033
Prior TIA	6.0%	6.5%	0.020
Prior intracerebral bleeding	0.6%	0.6%	0.001
Prior anemia	17.6%	8.0%	**0.290**
Prior major bleed	7.0%	4.9%	0.089
Prior GI bleed	6.7%	4.2%	**0.108**
COPD	7.9%	5.9%	0.081
Dementia	1.2%	1.3%	0.015
Frequent faller	3.3%	3.5%	0.007
Alcohol-related disease	2.3%	2.4%	0.005
Obesity	3.8%	3.6%	0.014
Thyroid disease	6.7%	6.3%	0.014
Liver disease	1.6%	1.0%	0.052
Venous thromboembolism < 6 mo	9.6%	4.3%	**0.211**
Platelet or coagulation disorders	1.9%	1.0%	0.074
Antithrombotic medication at OAC initiation			
NOAC	30.4%	26.8%	0.079
Previous platelet inhibitor	37.3%	39.3%	0.041
Cancer site			
Gastrointestinal	19.1%		
Pancreatic	1.0%		
Lung	6.8%		
Breast	9.1%		
Gynecological	4.9%		
Urological	35.6%		
Prostate	27.2%		
Intracranial	1.3%		
Hematological	10.7%		
Other	14.4%		
Metastasized a	9.2%		
Previous cancer treatment at OAC initiation			
Chemotherapy in hospital	3.0%		
Antitumoral drugs dispensed	13.5%		
Radiotherapy	5.1%		

Abbreviations: COPD, chronic obstructive pulmonary disease; GI, gastrointestinal; NOAC, nonvitamin K antagonist oral anticoagulant; OAC, oral anticoagulant; PCI, percutaneous coronary intervention; TIA, transient ischemic attack.

a Missing data on cancer stage: 43.1%. Standardized difference >10% in bold.

### Outcomes

During 1 year after OAC initiation and 308,505 contributed patient-years, 7,299 patients, of whom 2.8% had active cancer, suffered either an ischemic stroke or an intracranial bleeding. A total of 14,167 patients (4.8% with cancer) had bleedings, of which 25.8% were gastrointestinal, and 16.0% intracranial ( Supplementary Table S2 , available online only). The death rate was more than doubled in cancer patients, with 11.66 deaths per 100 patient-years (CI: 10.90–12.46), compared with noncancer patients (4.74 per 100 patient-years, CI: 4.66–4.81).

#### Cerebrovascular Events

**Fig. 1:**
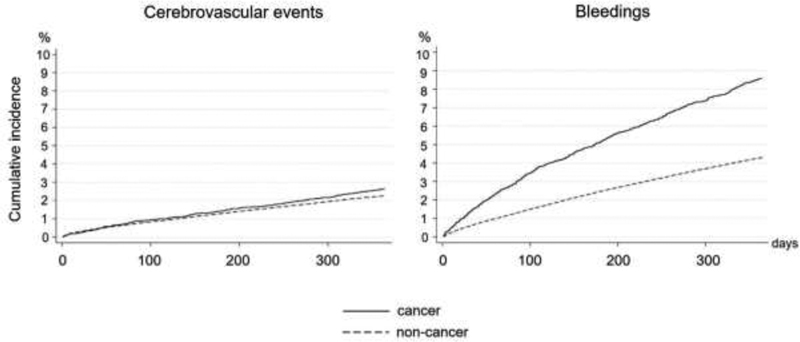
OAC-treated patients with atrial fibrillation, cancer versus noncancer patients: unadjusted cumulative incidences of cerebrovascular events and all bleedings during first year after OAC initiation, accounting for the competing risk of death. OAC, oral anticoagulant.

The cumulative incidences of patients with cerebrovascular events within the first year after OAC initiation were comparable between cancer patients (2.7%, CI: 2.3–3.0%) and noncancer patients (2.3%, CI: 2.2–2.3%) ( Fig. 1 ).

**Fig. 2:**
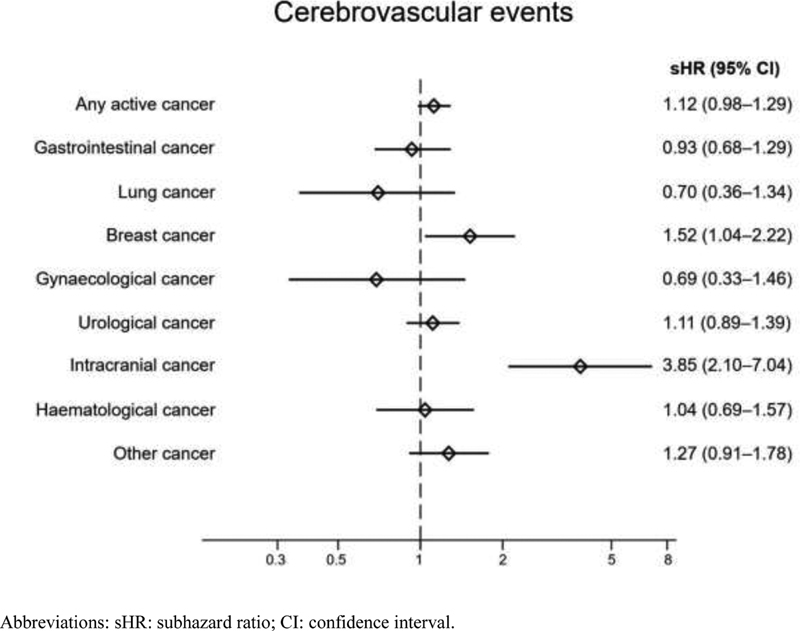
Adjusted risks for cerebrovascular events during the year following OAC initiation in patients with AF: cancer versus noncancer patients, accounting for the competing risk of death. AF, atrial fibrillation; OAC, oral anticoagulant.

Active cancer was not associated with a statistically significant higher risk for cerebrovascular events. Subgroup analyses showed higher risks only for patients with intracranial cancer and breast cancer ( Fig. 2 ). Breast cancer was, however, not associated with increased risk for ischemic stroke compared with no cancer (sHR: 1.30, CI: 0.81–2.09).

#### Predictors of Cerebrovascular Events

In addition to higher age, the composite prior ischemic stroke/transient ischemic attack/peripheral arterial emboli (sHR: 2.26, CI: 2.15–2.37) and prior intracerebral bleeding (sHR: 2.11, CI: 1.77–2.52) were the cofactors which showed the strongest associations with future cerebrovascular events among all studied patients. Other predictors were impaired kidney function, diabetes, hypertension, frequent falls, vascular disease, and more recent year of OAC initiation. Treatment with NOACs instead of warfarin was associated with a lower risk of cerebrovascular events (sHR: 0.78, CI: 0.73–0.83) ( Supplementary Table S3 ).

#### Bleedings after OAC Initiation

**Fig. 3:**
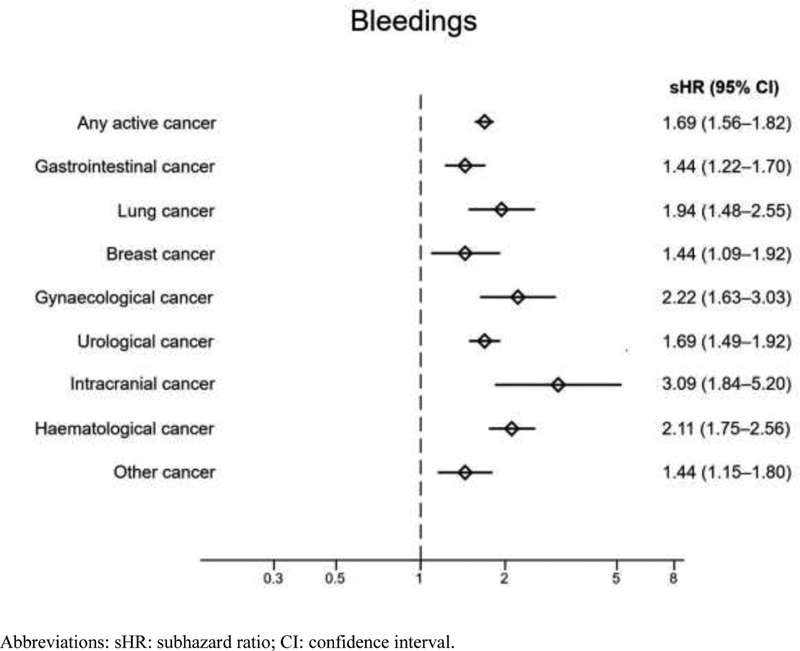
Adjusted risks for all bleedings during the year following OAC initiation in patients with AF: cancer versus noncancer patients, accounting for the competing risk of death. AF, atrial fibrillation; OAC, oral anticoagulant.

The cumulative incidence of patients with bleedings after OAC initiation was higher among cancer patients (8.6%, CI: 8.0–9.2%) than among noncancer patients (4.3%, CI: 4.2–4.4%) ( Fig. 1 ). Cancer patients regardless of cancer location had higher risk of bleedings than noncancer patients ( Fig. 3 ). Specifically, gastrointestinal bleedings were more common not only among patients with gastrointestinal cancer, but also among those with urological and hematological cancers, compared with noncancer patients. The overall increased risk for intracranial bleedings among cancer patients compared with noncancer patients was driven by individuals with intracranial or breast cancer ( Supplementary Table S7 , available online only).

Despite the higher risk of bleedings, active cancer was not associated with fatal hospital-treated bleedings overall (sHR: 1.17, CI: 0.80–1.70), neither with fatal intracranial bleedings (sHR: 1.13, CI: 0.71–1.82), accounting for the competing risk of death owing to other causes.

Besides active cancer, other factors independently associated with bleedings after OAC initiation were anemia, alcohol-related disease, prior major bleedings, liver disease, and heart failure. Others were shared with the primary outcome of cerebrovascular events; higher age, impaired kidney function, frequent falls, vascular disease, hypertension, diabetes, prior ischemic stroke/transient ischemic attack/peripheral arterial emboli, and later year of OAC initiation were associated with increased risk; NOAC treatment was associated with lower risk regarding all studied bleeding endpoints than warfarin ( Supplementary Tables S3–S6 , available online only). Exclusion of patients with CHA _2_ DS _2_ -VASc score 0 did not change results.

## Discussion

We have previously shown that AF patients both with and without cancer benefit from OACs, compared with no treatment. 4 In the present study, again using nationwide register data covering all individuals with AF and cancer, we aimed to compare OAC-treated AF patients with and without cancer. Related to our previous observations, the main finding was that the net cerebrovascular benefit was similar among patients with and without active cancer although the overall bleeding risk was higher among those with cancer.

Cancer patients had a higher comorbidity burden, including cardiovascular and bleeding risk factors, and additionally doubled mortality. After adjustment for cofactors, and for the competing risk of death, our analyses showed that the risk for cerebrovascular events was similar for patients with and without cancer. Patients with brain tumors did not appear to benefit from OAC owing to higher risk for intracranial bleedings. A similar lack of net benefit was found for patients with breast cancer. The reason for this is unclear, but may be due to an increased propensity for bleeding brought about by an interaction between vitamin K antagonists and selective estrogen receptor modulators used to inhibit tumor growth 19 or generally more intense antitumoral treatment which we could not adjust for. In previous studies of OAC-treated AF patients with breast cancer, the bleeding risk did not differ compared with OAC-treated AF patients without cancer. 20 21 However, in contrast to these studies, we recorded more events and studied only active cancers, making direct comparisons with these studies difficult.

Patients with gastrointestinal, urological, and hematological cancers had an increased risk for gastrointestinal bleedings. Several cancer types have been previously described as prone to gastrointestinal bleedings when treated with anticoagulants, mostly due to local barrier disruption of the gastrointestinal tract, thrombocytopenia, and invasive procedures or treatments, 22 and our data thus corroborate these findings.

Our findings are largely consistent with the posthoc analyses of the ENGAGE AF-TIMI 48 trial on OAC-treated patients with AF, in which cancer was not associated with all-cause stroke, but with major bleedings. 23 In the posthoc analyses of the of ROCKET AF trial, results were overall similar, with the exception that the bleeding risk of cancer patients was not seen for the specific endpoints: increased bleedings in critical organs and bleedings requiring blood transfusions. 24 This discrepancy in results may be attributed to the relatively low number of outcome events, the exclusion of patients with a life expectancy under 2 years, and less precise definitions of active cancer than in the present study which includes patients who are often not regarded eligible for drug trials.

Regardless of cancer status, NOAC use instead of warfarin use appeared to be safer regarding all studied endpoints. The greater safety for NOACs over warfarin regarding intracranial bleedings is in line with a large meta-analysis by Cavallari et al, including the posthoc studies mentioned. 5

Our study has several limitations. First, selection bias could be introduced since patients eligible for OAC treatment are likely to be healthier and have longer life expectancy than patients not offered OAC treatment, but also because hospital-based data tend to select toward individuals with heavier comorbidity. The proportion of cancer patients with metastases or antitumoral treatment was rather low, indicating that patients included in the present study had a possibly better prognosis than the total population of cancer patients with AF. Due to high proportions of missing data on cancer stage, and lacking validation of the registration of antitumoral treatment, these factors were not used in the analyses. This could introduce both treatment bias and missed associations between drug–drug interactions and outcome events. However, we assumed a greater impact of cancer type than stage, and by making all comparisons between patients eligible for OAC treatment, the effects of confounding by indication were minimized.

The main goal with OAC treatment in patients with AF is to prevent ischemic stroke, which has high mortality and the potential to cause impairment of function and life quality as well as rising health and social care costs, balancing the increased risk of bleedings, especially intracranial. 6 7

Third, as some of the analyses were conducted for every subgroup, this resulted in fewer events and the risk of low power to detect significant differences. However, a statistically significant increased risk of cerebrovascular events was seen among patients with intracranial cancer, which constituted the smallest of the subgroups studied.

Fourth, restricting follow-up time could influence generalizability beyond 1 year of OAC treatment. On the other hand, it minimizes possible bias introduced by an intention-to-treat-like approach which could underestimate associations with treatment due to crossover, and diverging prognoses over time among individuals with cancer.

Fifth, although we found that active cancer was associated with an increased risk for hospital-treated bleedings in general, no association was seen with fatal bleedings, with the reservation that an underestimation of fatal bleedings could occur since only hospital-associated events were studied.

Finally, being an observational study without randomization or individual assessment of endpoints, we can only report associations and do not claim that our findings represent causal relationships. However, this nationwide real-world register-based data add important information to the still developing field of OAC treatment among AF patients with cancer.

## Conclusion

Related to our previous findings, this study shows that among AF patients started on OAC treatment, no significant difference in net cerebrovascular benefit was found between noncancer patients and cancer patients, except for those with intracranial or breast cancer. Awaiting interventional studies with special focus on AF patients with cancer, our study supports current AF guidelines on OAC treatment originally aimed for the general AF population. As to the risk of all bleedings including intracranial bleedings, NOACs seem to be a safer alternative than warfarin.

## Notes

**What Is Known about This Topic?**

- Current guidelines on atrial fibrillation do not address stroke prevention with oral anticoagulants in the presence of active cancer. Increased risk of bleeding among cancer patients makes clinical decisions about oral anticoagulants challenging.
- Atrial fibrillation patients with cancer benefit from oral anticoagulant treatment with reduced risk of ischemic stroke and lower mortality compared with no treatment.

**What Does This Paper Add?**

- In this nationwide cohort study of all patients with atrial fibrillation (2006–2017), patients with active cancer appeared to have similar net cerebrovascular benefit of oral anticoagulants to patients without cancer, despite an increased overall risk of nonfatal bleedings.
- In the setting of atrial fibrillation, nonvitamin K antagonists seem to be a safer alternative than warfarin regardless of cancer status.
