# Supplementary material for: Atrial Fibrillation, Oral Anticoagulants, and Concomitant Active Cancer: Benefits and Risks
Source: TH Open. 2021 Jun 1;5(2):e176–82. doi: 10.1055/s-0041-1728670 (PMC8169314; doi:10.1055/s-0041-1728670)
Supplement: Supplementary file 1 — Supplementary Material [file 10-1055-s-0041-1728670-s200081.pdf]

Supplementary Material

Supplementary Table S1 Variable definitions

Variables	ICD-10 code or procedure beginning with
Ischemic stroke	I63
Atrial fibrillation or flutter	I48
Heart failure	I110, I130, I132, I255, I42, I43, I50, K761
Hypertension	I10, I11, I12, I13, I14, I15
Ischemic heart disease	I20, I21, I22, I23, I24, I25
Diabetes mellitus	E10, E11, E12, E13, E14
End-stage renal disease or dialysis	N185, Z992, DR055, DR056, QF006
Vascular disease	I21, I22, I252, I70, I71, I72, I73
Transitory ischemic attack	G450, G451, G452, G453, G458, G459
Impaired kidney function	E102, E112, N18, N19, I120, I131, I132, T861, Z992, Z940, DR055, DR056, QF006
Anemia	D461, D462, D464, D50, D51, D52, D53, D55, D56, D57, D58, D59, D60, D61, D62, D63, D64, Z513, DR029, DR030, DR033, DR034
Intracerebral bleeding	I61
Intracranial bleeding	I60, I61, I62, S064, S065, S066
Gastrointestinal bleeding	I850, I983, K226, K250, K252, K254, K256, K260, K262, K264, K266, K270, K272, K274, K276, K280, K282, K284, K286, K290, K625, K661, K920, K921, K922
Major bleeding	I690, I691, I692, and codes for intracranial and gastrointestinal bleedings
Major or nonmajor clinically relevant bleeding	D629, I312, M250, R040, R041, R042, R048, R049, R58, H313, H356, H431, R31, N421, N938, N939, KCV22, DR029, and codes for intracranial and gastrointestinal bleedings
Platelet or coagulation dysfunction	D66, D67, D68, D69
Mitral valve stenosis	I050, I052, I342, Q232
Mechanical heart valve	FGE00, FJF00, FKD00, FMD00, T820, Z952
Chronic obstructive pulmonary disease	J440, J441, J448, J449
Dementia	F00, F01, F02, F03, G051, G300, G301, G308, G309, G310, G311, G312, G318
Frequent falls, hospitalization $\geq$2 times	W00, W01, W04, W05, W06, W07, W08, W10, W17, W18, W19, R296
Alcohol-related disease	E244, F10, G312, G621, G721, I426, K292, K70, K860, O354, T51, Y90, Y91, Z502, Z714
Obesity	E66
Thyroid disease	E00, E01, E02, E03, E890, E05
Liver disease	Z944, K70, K71, K72, K73, K74, K75, K76, K77, JJB, JJC
PCI with or without coronary stent implantation	Z955, FNG02, FNG05
Venous thromboembolism $<$6 mo	I26, I801, I802, I81, I822, I823, I828, I829, I676, K765
Cancer	C0-C9, except C44
Any gastrointestinal cancer	C15, C16, C17, C18, C19, C20, C21, C22, C23, C24, C25, C26
Pancreatic cancer	C25

 © 2021. The Author(s).

Supplementary Table S1 (*Continued*)

Variables	ICD-10 code or procedure beginning with
Lung or pleural cancer	C340, C341, C342, C343, C348, C349, C384
Breast cancer	C50
Gynecological cancer	C51, C52, C53, C54, C55, C56, C57
Any urological cancer	C61, C64, C65, C66, C67, C68
Prostate cancer	C61
Intracranial cancer	C70, C71
Hematological cancer	C81, C82, C83, C84, C85, C86, C88, C90, C91, C92, C93, C94, C95, C96
Chemotherapy in hospital	DT107, DT108, DT112, DT116, DT135
Antitumoral medication prescribed (ATC code)	L01, L02B, L03AX03, L03AX11, L03AX15
Radio therapy	DV007, DV027, DV067, DV069, DV070, DV071, DV072, DV144, AAG50, CKE60, ZV520
Vitamin K antagonist	B01AA01 (dicoumarol), B01AA03 (warfarin)
Nonvitamin K antagonist oral anticoagulant	B01AF01 (rivaroxaban), B01AF02 (apixaban), B01AF03 (edoxaban), B01AE07 (dabigatran)
Parenteral anticoagulant	B01AB04, B01AB05, B01AB10, B01AX05
Antiplatelet drug	B01AC04, B01AC05, B01AC06, B01AC07, B01AC22, B01AC24 B01AC30, N02BA01, N02BA51

Abbreviation: PCI, percutaneous coronary intervention.
Note: ICD-10: International Classification of Diseases 10th Revision, implemented in 1997 in Sweden.

Supplementary Table S2 Outcome events during the year following OAC initiation in patients with atrial fibrillation, non-cancer versus all/various types of cancer: Number of events, incidence rates per 100 patients-years

Patients group (N)	Cerebral events		All bleedings		Gastrointestinal bleedings		Intracranial bleeding	
	n	IR/100 patient-years (95% CI)	n	IR/100 patient-years (95% CI)	n	IR/100 patient-years (95% CI)	n	IR/100 patient-years (95% CI)
non-cancer (323 394)	7091	2.35 (2.30–2.41)	13 489	4.52 (4.45-4.60	3483	1.15 (1.11–1.19)	2186	0.72 (0.69-0.75)
any active cancer (8228)	208	2.85 (2.49-3.27)	678	9.59 (8.89-10.33)	172	2.36 (2.03–2.74)	81	1.10 (0.89-1.37)
gastrointestinal cancer (1572)	38	2.44 (1.78-3.35)	152	10.11 (8.63-11.85)	50	3.23 (2.45–4.26)	16	1.02 (0.62-1.67)
lung cancer (561)	9	1.88 (0.98-3.62)	55	12.03 (9.24-15.67)	6	1.26 (0.57–2.81)	36	0.63 (0.20-1.95)
breast cancer (748)	27	3.67 (2.52-5.35)	49	6.78 (5.12-8.97)	9	1.21 (0.63–2.33)	12	1.62 (0.92-2.85)
gynaecological cancer (407)	7	1.81 (0.86-3.79)	42	11.28 (8.34-15.27)	9	2.32 (1.21–4.46)	1	0.26 (0.04-1.81)
urological cancer (2929)	78	2.80 (2.24-3.49)	248	9.18 (8.11-10.40)	59	2.12 (1.64–2.73)	29	1.03 (0.72-1.49)
intracranial cancer (105)	11	10.28 (5.69-18.56)	16	15.22 (9.33-24.85)	3	2.77 (0.89–8.58)	8	7.34 (3.67-14.68)
haematological cancer (878)	23	2.75 (1.83-4.14)	113	14.17 (11.79-17.04)	22	2.63 (1.73–4.00)	7	0.83 (0.40-1.75)
other cancer(1182)	35	3.31 (2.38-4.61)	78	7.53 (6.03-9.40)	27	2.54 (1.74–3.71)	11	1.03 (0.57-1.86)

Abbreviations: CI, confidence interval; IR, incidence rate; OAC, oral anticoagulant.

 © 2021. The Author(s).

Supplementary Table S3 Factor association with cerebrovascular events, accounting for the competing risk of death

	Multivariable sHR (95% CI)	*p*-Value
Age ≥ 85 y[a]	2.80 (2.55–3.08)	<**0.001**
Prior ischemic stroke, TIA, or extracranial arterial embolism	2.26 (2.15–2.37)	<**0.001**
Age 75–84 y[a]	2.12 (1.94–2.30)	<**0.001**
Prior intracerebral bleeding	2.11 (1.77–2.52)	<**0.001**
Age 65–74 y[a]	1.44 (1.31–1.57)	<**0.001**
Impaired kidney function	1.20 (1.08–1.34)	**0.001**
Diabetes	1.20 (1.13–1.27)	<**0.001**
Hypertension	1.19 (1.13–1.26)	<**0.001**
Frequent falls	1.14 (1.02–1.26)	**0.016**
Active cancer	1.12 (0.98–1.29)	0.097
Vascular disease	1.07 (1.02–1.14)	**0.013**
Year of OAC initiation[b]	1.02 (1.01–1.03)	<**0.001**
Female sex	0.98 (0.93–1.03)	0.387
Heart failure	0.95 (0.89–1.00)	0.057
NOAC treatment[c]	0.78 (0.73–0.83)	<**0.001**

Abbreviations: CI, confidence interval; NOAC, nonvitamin K antagonist oral anticoagulant; sHR, subhazard ratio; TIA, transitory ischemic attack.
Note: *p*-Values < 0.05 in bold.
[a]Reference: Age < 65 years.
[b]Reference: 2005.
[c]Reference: warfarin treatment.

Supplementary Table S4 Factor association with bleedings, accounting for the competing risk of death

	Multivariable sHR (95% CI)	*p*-Value
Age ≥ 85 y[a]	2.80 (2.62–3.01)	<**0.001**
Age 75–84 y[a]	2.26 (2.13–2.41)	<**0.001**
Prior anemia	1.94 (1.85–2.04)	<**0.001**
Active cancer	1.69 (1.56–1.82)	<**0.001**
Impaired kidney function	1.66 (1.56–1.76)	<**0.001**
Age 65–74 y[a]	1.56 (1.46–1.66)	<**0.001**
Alcohol-related disease	1.50 (1.37–1.65)	<**0.001**
Prior major bleeding	1.42 (1.34–1.51)	<**0.001**
Liver disease	1.36 (1.19–1.54)	<**0.001**
Frequent falls	1.26 (1.18–1.35)	<**0.001**
Heart failure	1.24 (1.19–1.29)	<**0.001**
Vascular disease	1.20 (1.16–1.25)	<**0.001**
Hypertension	1.17 (1.13–1.22)	<**0.001**
Diabetes	1.17 (1.13–1.22)	<**0.001**
Prior ischemic stroke, TIA, or extracranial arterial embolism	1.06 (1.02–1.10)	**0.006**
Year of OAC initiation[b]	1.06 (1.05–1.07)	<**0.001**
Female sex	0.91 (0.88–0.94)	<**0.001**
NOAC treatment[c]	0.78 (0.74–0.81)	<**0.001**

Abbreviations: CI, confidence interval; NOAC, nonvitamin K antagonist oral anticoagulant; sHR, subhazard ratio; TIA, transitory ischemic attack.
Note: *p*-Values < 0.05 in bold.
[a]Reference: Age < 65 years.
[b]Reference: 2005.
[c]Reference: warfarin treatment.

 © 2021. The Author(s).

Supplementary Table S5 Factor association with gastrointestinal bleedings, accounting for the competing risk of death

	Multivariable sHR (95% CI)	*p*-Value
Age ≥ 85 y[a]	3.05 (2.66–3.51)	<**0.001**
Age 75–84 y[a]	2.46 (2.17–2.79)	<**0.001**
Prior major bleeding	2.29 (2.08–2.52)	<**0.001**
Prior anemia	1.92 (0.76–2.09)	<**0.001**
Alcohol-related disease	1.80 (1.52–2.12)	<**0.001**
Liver disease	1.75 (1.41–2.17)	<**0.001**
Age 65–74 y[a]	1.70 (1.49–1.93)	<**0.001**
Active cancer	1.63 (1.40–1.91)	<**0.001**
Vascular disease	1.34 (1.24–1.44)	<**0.001**
Heart failure	1.32 (1.22–1.42)	<**0.001**
Diabetes	1.24 (1.14–1.34)	<**0.001**
Hypertension	1.20 (1.12–1.29)	<**0.001**
Year of OAC initiation[b]	1.04 (1.02–1.05)	<**0.001**
Female sex	1.01 (0.94–1.08)	0.767
Prior ischemic stroke, TIA, or extracranial arterial embolism	0.99 (0.92–1.07)	0.804
NOAC treatment[c]	0.88 (0.80–0.96)	**0.007**

Abbreviations: CI, confidence interval; NOAC, nonvitamin K antagonist oral anticoagulant; sHR, subhazard ratio; TIA, transitory ischemic attack.
Note: *p*-Values < 0.05 in bold.
[a]Reference: Age < 65 years.
[b]Reference: 2005.
[c]Reference: warfarin treatment.

Supplementary Table S6 Factor association with intracranial bleedings, accounting for the competing risk of death

	Multivariable sHR (95% CI)	*p*-Value
Prior intracerebral bleeding	4.15 (3.26–5.30)	<**0.001**
Age ≥ 85 y[a]	3.22 (2.70–3.85)	<**0.001**
Age 75–84 y[a]	2.51 (2.14–2.94)	<**0.001**
Age 65–74 y[a]	1.71 (1.45–2.01)	<**0.001**
Frequent falls	1.52 (1.28–1.80)	<**0.001**
Prior ischemic stroke, TIA, or extracranial arterial embolism	1.42 (1.30–1.56)	<**0.001**
Impaired kidney function	1.39 (1.17–1.66)	<**0.001**
Active cancer	1.33 (1.06–1.66)	**0.012**
Hypertension	1.33 (1.21–1.46)	<**0.001**
Year of OAC initiation[b]	1.07 (1.05–1.08)	<**0.001**
Vascular disease	1.05 (0.95–1.16)	0.348
Diabetes	1.01 (0.91–1.13)	0.805
NOAC treatment[c]	0.62 (0.55–0.70)	<**0.001**
Female sex	0.79 (0.73–0.87)	<**0.001**
Heart failure	0.89 (0.80–0.98)	**0.023**

Abbreviations: CI, confidence interval; NOAC, nonvitamin K antagonist oral anticoagulant; sHR, subhazard ratio; TIA, transitory ischemic attack.
Note: *p*-Values < 0.05 in bold.
[a]Reference: Age < 65 years.
[b]Reference: 2005.
[c]Reference: warfarin treatment.

 © 2021. The Author(s).

Supplementary Table S7 Adjusted risks for all bleedings, gastrointestinal bleedings, and intracranial bleedings during the year following OAC initiation in patients with atrial fibrillation, non-cancer versus all/various types of cancer: Number of events, incidence rates per 100 patients-years, adjusted subhazard ratios accounting for the competing risk of death. Statistically significant *P*-values in bold

First year after OAC initiation	All bleedings *		Gastrointestinal bleedings **		Intracranial bleedings ***	
	**sHR (95% CI)**	***P*-value**	**sHR (95% CI)**	***P*-value**	**sHR (95% CI)**	***P*-value**
non-cancer	reference		reference		reference	
any active cancer	1.69 (1.56–1.82)	**<0.001**	1.63 (1.40–1.91)	**<0.001**	1.33 (1.06–1.66)	**0.012**
gastrointestinal cancer	1.44 (1.22–1.70)	**<0.001**	1.70 (1.28–2.26)	**<0.001**	1.22 (0.74–1.99)	0.435
lung cancer	1.94 (1.48–2.55)	**<0.001**	0.79 (0.35–1.77)	0.562	0.69 (0.22–2.14)	0.519
breast cancer	1.44 (1.09–1.92)	**0.012**	0.98 (0.51–1.90)	0.961	2.35 (1.33–4.16)	**0.003**
gynaecological cancer	2.22 (1.63–3.03)	**<0.001**	1.75 (0.90–3.38)	0.096	0.34 (0.05–2.43)	0.283
urological cancer	1.69 (1.49–1.92)	**<0.001**	1.59 (1.23–2.07)	**<0.001**	1.19 (0.82–1.72)	0.358
intracranial cancer	3.09 (1.84–5.20)	**<0.001**	2.30 (0.73–7.27)	0.156	8.59 (4.12–17.90)	**<0.001**
haematological cancer	2.11 (1.75–2.56)	**<0.001**	1.58 (1.04–2.41)	**0.034**	0.94 (0.45–1.98)	0.875
other cancer	1.44 (1.15–1.80)	**0.001**	1.95 (1.33–2.85)	**0.001**	1.23 (0.68–2.23)	0.486

Abbreviations: CI, confidence interval; OAC, oral anticoagulants; sHR, subhazard ratio.

Adjusted for heart failure, hypertension, age, diabetes, the composite prior ischemic stroke/transient ischemic attack/peripheral arterial emboli, vascular disease, sex, year of OAC initiation, and: * anemia, prior major bleeding, impaired kidney function, liver disease, alcohol related disease, frequent falls, and use of NOAC instead of warfarin; ** anemia, prior major bleeding, liver disease, alcohol related disease, and use of NOAC instead of warfarin; *** prior intracerebral bleeding, impaired kidney function, frequent falls, and use of NOAC instead of warfarin.

 © 2021. The Author(s).
